# Patients' Perspective of Comprehensive Parkinson Care in Rural Victoria

**DOI:** 10.1155/2020/2679501

**Published:** 2020-03-31

**Authors:** Robert Iansek, Mary Danoudis

**Affiliations:** ^1^Clinical Research Centre for Movement Disorders and Gait Comprehensive Parkinson Care Program, Parkinson's Foundation Centre of Excellence Kingston Centre, Monash Health, 400 Warrigal Road, Cheltenham, VIC 3192, Australia; ^2^Faculty of Medicine, Nursing and Health Sciences, School of Clinical Sciences, Monash University, 246 Clayton Road, Clayton, VIC 3168, Australia

## Abstract

**Methods:**

This descriptive study used a survey to explore health-care experiences. Questionnaires were mailed to participants living in rural Victoria. Eligibility criteria included having a diagnosis of PD or Parkinsonism and sufficient English to respond to the survey. The validated Patient-Centred Questionnaire for PD was used to measure health-care experiences. The questions are grouped accordingly under one of the 6 subscales or domains. Outcomes from the questionnaire included summary experience scores (SES) for 6 subscales; overall patient-centeredness score (OPS); and quality improvement scores (QIS). Secondary outcomes included health-related quality of life using the disease-specific questionnaire PDQ39; disease severity using the Hoehn and Yahr staging tool; and disability using the Movement Disorders Society-Unified Parkinson's Disease Rating Scale, part II.

**Results:**

Thirty-nine surveys were returned from the East Gippsland group and 68 from the rural group. The East Gippsland group rated significantly more positive the subscales “empathy and PD expertise,” *P*=0.02, and “continuity and collaboration of professionals,” *P*=0.01. The groups did not differ significantly for the remaining 4 subscales (*P* > 0.05) nor for the OPS (*P*=0.17). The QIS showed both groups prioritised the health-care aspect “provision of tailored information” for improvement. Quality of life was greater (*P* < 0.05) and impairment (*P*=0.012) and disability were less (*P*=0.002) in the East Gippsland group.

**Conclusion:**

Participants who received health care from the East Gippsland program had better key health-care experiences along with better QOL and less impairment and disability. Participants prioritised provision of information as needing further improvement.

## 1. Introduction

Parkinson's disease (PD) is almost a lifelong progressive illness without cure. It manifests a profound complexity and a cumulative morbidity over the person's lifetime and its elderly age group presentation adds to the burdens of age-related comorbidities. Additional consequences of PD for the individual and their family include significant personal losses, financial disruption, relationship issues, and social isolation [1]. The clinical management of PD presents as a daunting task. Various service delivery models have been advocated, including an integrated care approach, but there is no consensus on ideal care [2].

A survey by the European Parkinson's Disease Association (EPDA) showed large gaps in care delivery for PD [3]. The EPDA led the call for a client-focused approach to care. In this context the Comprehensive Parkinson Care (CPC) program was developed [4]. The CPC program has a holistic approach, dealing with all aspects of the condition. It uses evidence-based interventions, which include medical, surgical, rehabilitative, educational, supportive, and lifelong lifestyle provision. The program is a multidisciplinary service that provides health services to the client in the outpatient clinic, the client's home (outreach), and the inpatient setting. Clients can also access members of the multidisciplinary team by phone and through telehealth. The CPC program provides continuity of care, extending from the community to residential facilities and to acute care. All members of the multidisciplinary team have extensive training, knowledge, and experience and have high caseloads of clients with PD or other movement disorders. The CPC program empowers the client and family to make the relevant decisions suitable to their needs through frequent patient contact and providing access to information and advice. The CPC is preventative in nature rather than reactive and through repetitive contacts with clients, and constant reinforcement is able to mitigate against the ever-declining quality of life that the condition infers on individuals. Extensive publications support the validity of this model of care. The CPC also has its own rehabilitation program developed in house and supported by publications [5–9]. Its transdisciplinary nature enables very good team cohesion, cooperation of members, and consistency for clients.

In Australia, little information exists about the PD service options available. The last economic analysis by Deloitte Access Economics [10] on PD in Australia showed a significant increase in costs associated with health-care delivery, productivity loss, and loss to the individual. The gaps and inequities in healthcare for people with PD living in Australia, especially in rural communities, are not dissimilar to the European experience [10, 11]. A higher prevalence of PD in rural areas and poorer perceived quality of life of rural Australians with PD has been reported [12]. Poor health-related quality of life is related to depression, disease severity, and disability [13], but whether these factors may in turn be related to limited access to specialist PD services in rural communities is unclear.

At the request of local PD support organisations to improve access to specialist PD services in East Gippsland, a clinical liaison was established in 2012 between the Comprehensive Parkinson Care (CPC) program, Melbourne, and two service providers in East Gippsland Victoria (Bairnsdale Regional Health Service (BRHS) and Gippsland Lake Community Health). Clinics involving a multidisciplinary team of selected staff, who underwent training on rehabilitation based on the CPC model, were established at each site. Clients with PD were referred to the geographically appropriate site for allied health assessments and interventions. Admission for medication adjustments was made through Monash Health, with rehabilitation undertaken at BRHS under Geriatrician supervision. Currently, approximately 100 country clients are managed under this modified comprehensive care system.

The aim of this study was to explore the impact of receiving specialised Parkinson's care in a rural setting on the person's health-care experiences. The health-care experiences of a sample of the cohort of people living with PD in East Gippsland, who had received a modified CPC program, was compared to those who received standard care from other rural health-care services across Victoria.

## 2. Methods

This descriptive study used a survey to explore the health-care experiences of people with PD living in rural Victoria.

### 2.1. Participants

The East Gippsland group was recruited from the East Gippsland Movement Disorders Program (MDP), and the rural group from the Parkinson's Victoria rural support groups based in communities without PD specialist programs. Eligibility criteria included having a diagnosis of PD or a Parkinson-related disorder (PRD) and having sufficient English to understand and respond to the survey when English was not the person's first language.

This study was promoted to people with PD, or a PRD, who were current patients of the East Gippsland MDP by mailing them a letter of invitation to participate. Letters were mailed to 75 patients all of whom had their diagnosis of PD or PRD confirmed by the East Gippsland movement disorder specialist. People with PD living in other Victorian rural communities were recruited through 17 Parkinson's Victoria rural support groups. Pamphlets were distributed to group members by leaders at their regular meetings. The letters and pamphlets included information regarding the study and the study coordinator's contact details. Potential participants contacted the coordinator by phone or email to obtain a questionnaire. The coordinator asked all potential participants to confirm their diagnosis before mailing them the survey. The survey was anonymous with only the person's group allocation recorded on it. Surveys were sent by mail along with a stamped addressed envelope for return mail.

### 2.2. Measures

Participants' health-care experience was measured using the Patient-Centred Questionnaire for PD (PCQ-PD) [14]. This questionnaire was validated with Dutch and North American populations and was shown to be cross culturally valid [14, 15]. This current study used the Dutch version of the PCQ-PD, available in English, as it included questions about each health-care member involved in our multidisciplinary team. We wished to capture the client's health-care experiences for all team members who had provided care. The North American version of the questionnaire did not individualise each health-care worker rather the questions related to the generic title of health professional. Minor changes were made to the PCQ-PD following review of its content by three patients with PD and 5 health professionals with expertise in the management of PD. The changes improved clarity of the wording and ensured the questions were applicable to the Australian medical landscape. There were six questions in the Dutch version that were only asked about the neurologist and nurse, e.g., did your neurologist/nurse listen carefully to you. We elected to include these six questions for the physiotherapist, occupational therapist, speech pathologist, neuropsychologist, and social worker, as they were considered relevant to the evaluation of healthcare in Australia. There were a total of 134 questions, with an additional 17 questions capturing information on participants' characteristics. As in the Dutch version, 46 care aspects were allocated to the following six subscales: involvement in decision-making; provision of tailored information; accessibility of healthcare; empathy and PD expertise; continuity and collaboration of professionals; and emotional support. Response categories and scoring were applied as described previously [14]. Outcomes included (1) item experience scores (IES) with a maximum possible of 3 and lowest of 0, with 3 indicating the most positive experience; (2) subscale experience score (SES) for each of the 6 subscales; (3) overall patient-centeredness score (OPS), the mean of total SES scores; (4) item priority scores (IPS) with 0 not important and 3 extremely important; and (5) quality improvement score (QIS), where QIS = (3-IES) ∗ IPS and 0 = low improvement priority and 9 = high improvement priority. Participants prioritised 44 health-care experiences. For this study, where a priority question was repeated for more than 1 profession, the average score was calculated first and then the QIS.

The following validated PD measures were included with the survey: (1) Self-reported Hoehn and Yahr (H&Y) stage, a measure of impairment [16]; (2) Parkinson's disease Questionnaire 39 (PDQ39) to quantify health-related quality of life (HRQOL) [17]; (3) Movement Disorders Society-Unified Parkinson's Disease Rating (MDS-UPDRS) Part II (motor experiences of daily living) to assess disability [18]; and (4) Non-Motor Symptom Questionnaire (NMS-Quest), number of nonmotor symptoms [19]. Demographic information was also collected.

### 2.3. Statistical Analysis

SPSS software (IBM V 24) was used to analyse all data. Group characteristics involving categorical variables were reported as frequencies and percentages; continuous variables were reported as means and standard deviations (SD) or medians and range. Categorical variables were compared by group using *x*^2^ tests for independence, using Yates' Correction for Continuity output or Fisher's Exact Probability Test. Independent samples *t*-tests were used to estimate the mean differences between groups for continuous variables.

Based on a previous study that used the PCQ-PD, the aim was to recruit 50 participants per group [15].

## 3. Results

Recruitment for the East Gippsland group commenced in December 2016 and finished in February 2017 and that for the rural group commenced in May 2017 and was completed in December 2017. A total of 107 surveys were returned, with 39 allocated to the East Gippsland group, a response rate of 51%, and 68 to the rural group. The participation rate for the rural group could not be determined as it was not feasible to record the number of pamphlets distributed by Parkinson's Victoria at their rural support groups. All returned surveys were checked for the number of experience questions answered and only those with ≥50% questions answered were included in the analysis [15]. All 107 surveys met this criterion.

In these two groups of older adults, mean age 72.6 years (range 49–88), over 95% reported they were diagnosed with idiopathic PD (Table 1). The Hoehn and Yahr stage was significantly lower for the East Gippsland group, indicating milder impairment (*P*=0.012). The East Gippsland group rated the healthcare they received for their PD significantly higher (*P*=0.01).

### 3.1. Primary Outcome: PCQ-PD

The OPS did not differ significantly between groups (*P*=0.17); however, health-care experiences were rated significantly higher, or more positive, by the East Gippsland group for the subscales “empathy and PD expertise” (*P*=0.02) and “continuity and collaboration of professionals” (*P*=0.01) (Table 2). Groups did not differ significantly for the remaining 4 subscales. The QIS results (Table 3) indicated both groups prioritised the same top five care aspects for quality improvement with no significant difference (*P* > 0.05) (Table 3). All five items were from the “provision of tailored information” subscale. The top priority item for quality improvement rated by both groups was “you are informed about what your health professionals discuss with each other regarding your PD treatment.” The rural group gave significantly higher priority to improving aspects of “emotional support” (*P*=0.02) and “continuity and collaboration of professionals” (*P* ≤ 0.01).

### 3.2. Secondary Outcomes

The summary score index of the PDQ39 and scores for four of the eight individual dimensions were significantly lower in the East Gippsland group (*P* < 0.05), indicating better health-related quality of life (Table 4). Disability, measured by the MDS-UPDRS Part II was significantly milder (*P*=0.002) in the East Gippsland group. The motor scores suggested the East Gippsland group had mild disability and the rural group moderate disability [20]. Nonmotor symptoms were common in both groups.

## 4. Discussion

This survey showed that those receiving care from the CPC program rated aspects of their health-care experience higher than those treated with standard care. Continuity and collaboration of professionals along with empathy were rated significantly higher in the East Gippsland group. The overall patient-centeredness score was greater than the midpoint of the scale for both groups, seen as positive with such surveys [21]. Both groups rated their experience of “provision of information” low. The rural group rated aspects of emotional support and continuity and collaboration as having a significantly higher improvement priority compared to East Gippsland. Items from provision of tailored information subscale were rated high for improvement potential by both groups. Heath-related quality of life was better and impairment and disability less in the East Gippsland group.

Patient-centred care that tailors care to the needs of the individual is critical in the management of PD [22]. Expert multidisciplinary PD teams that provide continuity of care and work collaboratively are essential for patient-centred care and are what people with PD want [21, 22]. The more positive experience of “continuity and collaboration” by the East Gippsland group suggests the CPC program is providing a critical aspect of healthcare that patients prefer and value. The rural group rated this aspect of care as high priority for improvement.

Being listened to, having things explained in a way that is easy to understand, and clinicians being competent in regards to treatment of the person's PD are aspects of care that people with PD want clinicians to provide [22]. Clinicians need to have PD training, work with other clinicians expert in managing PD and maintain a high PD-specific case load if they are to provide these desired aspects of care [2]. The CPC program does this and its success in meeting patient needs is supported by the finding that patients from the program had a greater positive experience of empathy and expertise compared to those receiving standard care.

Both groups had a moderate OPS score which did not differ significantly between them. The small number of participants in the East Gippsland group may have contributed to this lack of significance; however, the significant findings for some subscales suggests other factors other than sample size may have contributed to this finding.

Both groups indicated quality improvement for “provision of information” was a priority. Interestingly, the Dutch and American experiences showed similar findings from their surveys [14, 15]. The ability of people with PD to understand and remember what health professionals tell them is dependent on various factors such as cognitive function and how the information is presented [22]. The CPC program prioritises keeping patients and their carers informed about their PD; however, these results suggest this aspect of care needs reviewing.

Positive health-care experiences are associated with better HRQOL and improved physical, emotional, and social outcomes in PD [21]. Though this study did not monitor the changes in function and HRQOL over time, the higher rating of received healthcare, the better health-care experiences, and the lower priority for improvement of key aspects of care experienced by the East Gippsland group, suggest the CPC program contributed to the better HRQOL and disability reported by this group compared to the rural group.

### 4.1. Limitations

The majority of responders were at a mild-to-moderate stage of PD which limits the application of these findings to those with later stage PD. The sample size for the East Gippsland group was smaller than the rural group; however, significant differences between groups were found. The Hoehn and Yahr staging tool is typically administered by a clinician, but as there was no face-to-face interaction with participants, they were asked to self-rate their disease stage. Any inaccuracies were expected to be similar between groups. The PCQ responses provided other information about possible improvements in care provision, the findings of which were beyond the scope of this paper. The similarity in outcomes for the PCQ across countries with differing models of care suggests a limitation of the design of the questionnaire itself, which may need further investigation.

## 5. Conclusion

The East Gippsland-modified CPC model appears to have resulted in better patient-reported outcome measures (PROMs). The HRQOL index score revealed a difference of four times the meaningful clinical significance, suggesting a robust difference between the groups. Disease severity, a key predictor of HRQOL, is unlikely to explain this difference in HRQOL as both age and duration of disease were the same for both groups. The emotional well-being dimension of the PDQ39 did not differ between groups suggesting mood also did not explain HRQOL differences. Impairment or Hoehn and Yahr stage, disability, and ADL dimension of PDQ39 were significantly better in the East Gippsland group, suggesting better medical management. The higher rating of healthcare received by the East Gippsland group supports these findings. These findings are consistent with the recommendations from the EPDA [3] and the Parkinson charter, which recommends that PD care include trained and experienced health-care professionals working in concert with a Parkinson specialist.

The disparity between the PROMs and the PCQ-PD findings suggest they address differing aspects of patient care. Both are important as both constitute quality of care and both need to be reviewed and addressed regularly to ensure optimum outcome for clients.

Overall, the modified CPC program has resulted in a better HRQOL for the East Gippsland group, through perhaps better symptom control. However, from the clients' perspective, care delivery still needs further improvement. The ability to improve care to rural Australians with PD requires very limited input as this modified approach utilised education and training of existing teams in the rural setting supported by regular expert advice. Such an approach should be considered more widely given key movement disorders centres exist in each capital city.

## Figures and Tables

**Table 1:** Demographic characteristics of survey respondents for the East Gippsland and rural groups.

Characteristics	East Gippsland group (*n* = 39)	Rural group (*n* = 68)	*Pvalue*
Age, mean (SD) (years)	73.7 (7.2)	72.0 (7.4)	0.24
Sex: Male, *n* (%)	28 (73.7)	39 (57.4)	0.14
Female, *n* (%)	10 (26.3)	29 (42.6)	*X* ^2^ (1, *n* = 106)
			= 2.1^a^
Diagnosis: Idiopathic PD, *n* (%)	38 (97.4)	65 (95.5)	1.0^b^
Parkinsonism, *n* (%)	1 (2.6)	3(4.5)	
PD duration, mean (SD) (years)	6.7 (5.3)	7.3 (5.4)	0.64
Hoehn and Yahr median (IQR)	1 (1.4)	3 (1 5)	0.01
HY1, *n* (%)	19 (51.4)	10 (14.7)	
HY2, *n* (%)	1 (2.7)	17 (25.0)	
HY3, *n* (%)	15 (40.5)	35 (51.5)	
HY4, *n* (%)	2 (5.4)	5 (7.4)	
HY5, *n* (%)	0	1 (1.5)	
Rating received healthcare, *n* (%)			0.01^b^
Excellent	14 (35.9)	15 (22.1)	
Very good	19 (48.7)	21 (30.9)	
Good	5 (12.8)	15 (22.1)	
Fair	1 (2.6)	11 (16.2)	
Poor	0	6 (8.8)	
Self-reported physical health, *n* (%)			0.06^b^
Excellent	0	6 (8.8)	
Very good	13 (33.3)	11 (16.2)	
Good	17 (43.6)	26 (38.2)	
Fair	7 (17.9)	22 (32.4)	
Poor	2 (5.1)	3 (4.4)	
Self-reported mental health, *n* (%)			0.53^b^
Excellent	4 (10.3)	4 (6.0)	
Very good	13 (33.3)	27 (40.3)	
Good	15 (38.5)	22 (32.8)	
Fair	6 (15.4)	14 (20.9)	
Poor	1 (2.6)	0	
Living situation, *n* (%)			0.66^b^
Alone	5 (12.8)	8 (11.8)	
With partner/spouse	31 (79.5)	53 (77.9)	
With partner plus relatives	1 (2.6)	5 (7.4)	
With friends	1 (2.6)	0	
Supported accommodation	1 (2.6)	2 (2.9)	0.08^b^
Caregiver, *n* (%)			
None	17 (43.6)	15 (22.4)	
Spouse/partner	21(53.8)	48 (71.6)	
Other relative	0	1 (1.5)	
Paid caregiver	1 (2.6)	3 (4.5)	
Education completed, *n* (%)			0.28^b^
High school level ≤ year 11	22 (56.4)	28 (41.8)	
High school level year 12	3 (7.7)	9 (13.4)	
Technical or TAFE	7 (17.9)	21(31.3)	
University degree or higher	7 (17.9)	9 (13.4)	

*n* = number of participants/data points; % = percentage; IQR = interquartile range; TAFE = technical and further education; a = Yates' correction; *b* = Fisher's exact test.

**Table 2:** Subscale experience scores (SES) for 6 domains mean (SD).

Experience subscales	East Gippsland group (*n* = 39)	Rural group (*n* = 68)	*Pvalue* (95% CI)
OPS	1.9 (0.5)	1.8 (0.6)	0.17^a^
			−0.06, 0.36
Involvement in decision-making	1.9 (0.7)	1.8 (0.7)	0.35
			−0.16, 0.44
Provision of tailored information	1.3 (0.6)	1.4 (0.5)	0.28
			−0.33, 0.10
Accessibility of healthcare	2.2 (0.6)	2.3 (0.5)	0.45
			−0.40, 0.18
Empathy and PD expertise	2.6 (0.4)	2.4 (0.6)	0.02^a^
			0.04, 0.43
Continuity and collaboration of professionals	2.1 (0.8)	1.6 (0.9)	0.01
			0.11, 0.82
Emotional support	1.8 (0.9)	1.5 (1.0)	0.12
			−0.09, 0.77

OPS = overall patient-centeredness score (range 0–3) and SES = subscale experience scores (range 0–3), higher scores indicate more positive experience. CI = confidence intervals; a = equal variances not assumed.

**Table 3:** Quality improvement scores for top priority items.

Items	Subscale	QIS East gippsland group, mean (SD)	QIS Rural group, mean (SD)	*Pvalue,* 95% CI
Informed about what health professionals discussed with each other	Provision of tailored information	4.7	5.2	0.40
		(3.0)	(3.1)	−1.85, 0.74
Knowledge about advanced PD treatment options	Provision of tailored information	4.6	4.1	0.47^a^
		(2.4)	(3.3)	−0.94, 1.86
Knowledge about alternative health therapies	Provision of tailored information	4.5	4.4	0.90
		(2.9)	(3.0)	−1.22, 1.38
Know about treatment options for PD provided by different clinicians	Provision of tailored information	3.9	4.5	0.27
		(2.5)	(2.5)	−1.57, 0.44
Contacted by health professional after starting new PD medication regimen	Provision of tailored information	3.7	4.5	0.28
		(3.5)	(3.1)	−2.30, 0.65
Support from health professionals to cope with consequences of PD	Emotional support	2.6	4.3	0.02^a^
		(1.9)	(3.1)	−2.00, −0.34
Have one person assigned to you whom you can contact	Continuity and collaboration of professionals	2.2	4.2	0.01
		(3.5)	(3.5)	−3.55, −0.46
Have one health professional with whom to make most important decision regarding PD	Continuity and collaboration of professionals	1.5	4.1	0.001^a^
		(3.2)	(3.8)	−4.09, −1.02

QIS = quality improvement scores (range 0–9), higher scores indicate higher priority for improvement; CI–confidence intervals; a = Equal variances not assumed.

**Table 4:** Parkinson's disease-related outcome measures.

Variables	East Gippsland group, *n* = 39 (mean (SD))	Rural group, *n* = 68 (mean (SD))	*P* value
PDQ39			
SI	23.1 (15.0)	31.7 (15.2)	0.01
Mobility	27.2 (26.6)	43.8 (28.4)	0.006
ADL	21.3 (22.2)	32.8 (23.0)	0.01
Emotion	24.8 (19.0)	30.1 (19.5)	0.18
Stigma	20.7 (22.0)	22.7 (20.5)	0.65
Social	9.6 (12.7)	18.8 (21.2)	0.007^a^
Cognition	29.6 (17.0)	35.9 (20.5)	0.12
Communication	22.4 (19.3)	34.5 (23.7)	0.009
Discomfort	32.2 (21.3)	46.0 (22.8)	0.003
MDS-UPDRS Part II M-EDL	11.5 (7.6)	17.0 (8.6)	0.002
NMSQuest	11.2 (5.4)	12.9 (5.5)	0.15

PDQ39 SI = Parkinson's disease Questionnaire 39 summary index, higher scores indicate poorer quality of life; ADL = activities of daily living; MDS-UPDRS = Movement Disorders Society-Unified Parkinson's Disease Rating Scale, higher scores indicate poorer experiences of daily living; M-EDL = motor aspects of experiences of daily living; NMSQuest = nonmotor symptom questionnaire, higher scores indicate greater number of nonmotor symptoms; a = equal variances not assumed.

## Data Availability

The data files are held by the Monash Health Clinical Research Centre for Movement Disorders and Gait as per the Monash Health Human Research Ethics Committee requirements.
